# A Dream Realized

**Published:** 1888-01

**Authors:** 


### A DREAM REALIZED—FOREWARNED FOREARMED.

A few years ago a man named Bronson, who was an agent for a big seed house, was travelling through Tennessee making collections for his house. He had to visit many towns off the railroads, and in such cases, he secured a horse and buggy or rode horseback. One night, according to the Mobile Register, after he had finished his business in Chattanooga, he made ready for a horseback ride of fifteen or twenty miles the next day. Upon retiring to his room for the night he sat down to smoke a cigar.

He was neither overtired nor sleepy, but, after smoking a few minutes, he had what he termed, a vision. He was riding over the country on horseback, when at the junction of two roads he was joined by a stranger. He saw this man as plainly as one man can see another in broad daylight, noting the color of his hair and eyes and taking particular notice of the fact that the horse, which was gray in color, had a "y" branded on his left shoulder.

The two rode along together for a mile or more, and then came to a spot where a tree had blown down and fallen across the narrow highway. They turned into the woods to pass the spot, he in advance, when he saw the stranger pull a pistol and fire at his back. He felt the bullet tear into him, reeled and fell from his horse, and was conscious when the assassin robbed him and drew his body further into the woods. He seemed to see all this, and yet at the same time, knew that he was dead. His corpse was rolled into a hollow and covered with brush, and then the murderer went away and left him alone.

In making an effort to throw off the brush, the dead man came to life; that is, the agent threw off the spell and awoke himself. His cigar had gone out, and, as near as he could calculate, he had been unconscious, as you might call it, for about fifteen minutes. He was deeply agitated, and it was some time before he could convince himself that he had not suffered any injury. By-and-bye he went to bed and slept soundly, and next morning the remembrance of what had happened in his vision had almost faded from his mind.

Luckily for Bronson, he made some inquiries at the livery stable as he went for his horse, and he was told that it was a lonely road, and that he would be prudent to go armed. But for this, he would have left his revolver in his trunk at the hotel. He set out on his journey in good spirits, and found the road so romantic and met horsemen going to town so often, that he reached the junction of the roads without having given a serious thought to his vision.

Then every circumstance was recalled in the most vivid manner.

He was joined there by a stranger on a gray horse, and man and beast tallied exactly with those in the vision. The man did not, however, have the look or bearing of an evil minded person. On the contrary, he seemed to be in a jolly mood, and he saluted Bronson as frankly as an honest stranger would have done. He had no weapons in sight, and he soon explained that he was going to the village to which Bronson was bound, on business connected with the law.

The agent could not help but feel astonished and startled at the curious coincidence, but the stranger was so talkative and friendly that there was no possible excuse to suspect him. Indeed, as if to prove to his companion that he meditated no evil, he kept a little in advance for the next half hour. Bronson's distrust had entirely vanished, when a turn in the road brought an obstruction to view. There was a fallen tree across the highway! This proof that every point and circumstance in the vision was being unrolled before his eyes, gave the agent a great shock. He was behind the stranger, and he pulled his revolver and dropped his hand beside the horse to conceal it.

"Well, well!" said the man, as he pulled up his horse. "The tree must have toppled over this morning. We'll have to pass around it to the right."

Bronson was on the right. The woods were clear of underbrush, and naturally enough, he should have been the first to leave the road, but he waited.

"Go ahead, friend," said the stranger, and as if the words had been addressed to the horse, the animal which the agent bestrode started up.

Bronson was scarcely out of the road before he turned in his saddle. The stranger had a pistol in his right hand. What followed could not be clearly related. Bronson slid from the saddle as a bullet whizzed past him, and a second later returned the fire. Three or four shots were rapidly exchanged, and then the would-be-murderer, uttering a yell to show that he had been hit, wheeled his horse to gallop off. He had not gone ten rods when the beast fell under him, and he kicked his feet from the stirrups and sprang into the woods and was out of sight in a moment. The horse had received a bullet in the throat and was dead in a few minutes.

As a matter of course, Bronson put the case in the hands of the proper officials, but the horse could neither be identified nor the man overhauled. It was agreed that he was an entire stranger in that locality, and that, while he did not know Bronson nor the business he was engaged in, he was ready to commit a cold-blooded murder and take his chances of finding a fat wallet to repay him.

